# Solar Cookers and Dryers: Environmental Sustainability and Nutraceutical Content in Food Processing

**DOI:** 10.3390/foods10102326

**Published:** 2021-09-30

**Authors:** Chiara Battocchio, Fabio Bruni, Giovanni Di Nicola, Tecla Gasperi, Giovanna Iucci, Daniela Tofani, Alessandro Varesano, Iole Venditti

**Affiliations:** 1 Dipartimento di Scienze, Università degli Studi Roma Tre, Via della Vasca Navale 79, 00149 Rome, Italy; chiara.battocchio@uniroma3.it (C.B.); fabio.bruni@uniroma3.it (F.B.); tecla.gasperi@uniroma3.it (T.G.); giovanna.iucci@uniroma3.it (G.I.); iole.venditti@uniroma3.it (I.V.); 2 Dipartimento di Ingegneria Industriale e Scienze Matematiche, Università Politecnica delle Marche, Via Brecce Bianche, 60131 Ancona, Italy; 3 Helio.it, Via Laurentina 749A, 00143 Rome, Italy; ing.alessandro.varesano@gmail.com

**Keywords:** solar cooker, solar dryer, food processing, nutraceuticals, antioxidants, vitamins

## Abstract

This work reviewed the state of the art concerning solar cookers and dryers used in food processing. The general description of solar cookers and dryers was presented, with a specific attention to the equipment where the cooking takes place with the contribution of the direct sunlight. Some insight about the history of design and development of devices that use solar light to process food were provided. The possibility to store the heat produced by solar light using Phase Change Materials was analyzed. Moreover, some “case-studies” were revised and discussed, in which solar light is efficiently used to dry or cook food, focusing on the quality of the food in terms of nutraceuticals content. The analyzed literature points out the necessity for further research about the effects produced by direct solar rays on different foods. The reliable data on this aspect will allow assessment of the quality of food transformation by solar cookers and dryers, adding a strong incentive to the development of such devices, up to now primarily motivated by energy-saving and environmental issues.

## 1. Introduction

In the last decade, research has given a lot of space and attention to the development of materials, instruments and production processes that take into account and allow environmental protection and sustainability [1,2,3,4,5,6]. Certainly, the development of new technologies and materials is moving in this green direction, as evidenced by the fervent scientific production on this issue. Furthermore, the use of clean and renewable energy represents a new possibility of development for many companies increasingly involved in the ecological transition. In this context, solar energy is enjoying great success, both in terms of investments for research and development, and also in terms of facilities for companies that are able to modify and/or implement processes using this source of energy [7,8,9,10].

In fact, solar energy is clean and safe and guarantees use without negative impact on the environment and society. Historically, solar energy has always been present in daily activities, such as cooking, drying food and clothes and heating water. Moreover, following innovative research activities, new fields of application of solar energy have been developed, which today are also used for drying, steam and energy production, water distillation and desalination, heating, cooling and refrigeration [11]. The solar food cookers (SCs) and dryers (SDs), even if they require an upfront expense, provide better taste and safe, marketable nutritious food. The food industry is also fully involved in these applications, both in the production and in the processing of food (cooking and dehydration) [12,13,14,15,16,17]. Numerous studies have shown that solar cooking and drying can be an effective means of food preservation as the product is completely protected from rain, dust, insects and animals. However, some obstacles still need to be overcome for solar cooking and drying to become a more widespread technology. Although a lot of research has been done in the past few decades, only a limited number of suitable SCs and SDs are commercially available that can be used by farmers or small-scale industries. Furthermore, there is still a lack of knowledge on how to handle foods, such as vegetables, fruit, meat, fish, etc. correctly to ensure a tasty, high quality product and minimize losses. So far energy saving has been the main driver for SCs and SDs. However there is also a second reason that has been analyzed and that still needs more research: the quality of the food transformation during the process.

This review deals with the SCs and SDs used for food processing. For this topic, more than 100 articles have been examined, mainly from the last decade. Each section discusses previous research and highlights future opportunities. Several cross-cutting themes emerge from the literature and suggest future directions on the use of solar energy for food processing. The need for fundamental research on SCs and SDs is justified by its impact, for instance on food, on transition from laboratory to large-scale integrated systems, on increased economic profitability and on increased efficiency in the use of resources. Therefore, a review of the technology that uses solar energy for food processing is presented, treating the SCs and the SDs together. This work aims above all to be a help and a stimulus for the scientific community that is confronted on these issues: by comparing the two systems in a single review. In fact, their strengths and criticalities, often similar, are underlined. Furthermore, it is highlighted how it is possible and desirable to improve current performance accompanied by a careful assessment of the quality of the food processed in this way. All this considered, that there is a strong potential for this technology, not only in developing countries, but also in richer countries where attention to eco-sustainable industrial processes is increasing.

## 2. The History of Solar Cookers and Dryers

It is not only in recent years that solar light has been used to cook or dry food. The greenhouse can be considered the ancestor of the SC box and SDs, as both retain solar heat within a confined space. From this concept, in 1767, Horace-Bénédict de Saussure, a Swiss scientist, started to build the first real SC in history, with the aim of studying the collection of heat inside glass boxes. Another figure certainly fundamental for the development of the study of solar thermal energy was the French inventor Augustin Mouchot (1825–1912) who invented a glass boiler that, when exposed to the sun brought the water to boiling, whose steam then powered a small steam engine [18,19].

Around the end of the nineteenth century the American Aubrey Eneas, retracing the work of Mouchot, built a large parabolic reflector in the USA and set up the first company involved in the production of solar devices. Frank Shuman (1862–1918) established the Sun Power Company and built the world’s first solar power plant in Maadi, Egypt, consisting of parabolic mirrors to power a 60–70 hp engine capable of pumping 23,000 L of water per minute from the Nile to the adjacent cotton fields [20]. Unfortunately, even the results of Shuman’s research did not enter the market, since they proved to be economically not competitive with respect to coal and oil which, still having a low price, remained the sources of cheaper energy on the market.

In the same period, Alessandro Annibale Battaglia (1842–unknown) was the first to realize that, to concentrate much more energy, it is required to separate the receiver (the oven box) from the mirrors [21].

An important contribution to modern solar cooking was made by Maria Telkes (1900–1996) [22]. During the 1920s she invented a plastic bag still capable of producing, by sun heating, a few liters of fresh water from sea brine for castaways. In 1948, Maria Telkes directed the construction of the first solar-heated experimental house, followed by thermoelectric generators for space and terrestrial uses. From 1959 she dedicated herself to the construction of a solar kitchen, a structure for outdoor use consisting of a central body in which food was inserted and a series of reflective aluminum panels arranged to capture the heat of the sun. The reflectors popularly known as the “Telkes kitchen” were among the best solar cookers and developed a temperature of 225 °C.

Another pioneer of solar energy technology was George Löf (1913–2009), director of the Industrial Research Institute at the University of Denver, Colorado. In 1950, he experimented with a parabolic SC which he nicknamed the “Umbroiler”, as its shape resembled that of an umbrella [23]. This project proved to be economically unsuccessful for the time, although he subsequently managed to distribute other models of SCs in various third world countries thanks to UNESCO.

A further impetus to the discovery of solar cooking came during the 1980s, due to the incipient oil crisis, with considerable experimentation in Europe and the USA. It is no coincidence that groups such as ULOG (Switzerland), EG Solar (Germany) and Solar Cookers International (USA) were born in those years. It was also in the 1980s that, Barbara Kerr experimented with the efficiency of various types of SCs, especially wall-mounted SCs and solar panel ovens, and it is this last type that was very successful.

In the same period, a large event was held to raise awareness of the population towards solar cooking in the Bolivian highlands, an area of the world heavily subject to shortage of timber and deforestation. The event was organized by the Pillsbury Corporation and Meals for Millions, which held cooking demonstrations and lectures on building SCs with local materials. In 1988, the Pillsbury Corporation, in partnership with Foster Parents (now Save the Children), sponsored a similar project in Guatemala. These were the first international projects in the field of solar cooking, which then gave way to other similar projects that continue to this day. 

Finally, the first mass-utilization of solar cookers was in India, where every year a special day with a huge number of students from several schools is organized. During this event, a practical use of solar cookers is shown.

The history of properly defined dryers is more recent. Solar drying has been a simple and inexpensive way to process and store food since ancient times. This treatment removes water or moisture present in various ingredients and prevents fermentation. Everitt and Stanley in 1976 made the first solar dryer that avoided the problems of drying in the open sun [24]. It was a simple box-shaped unit that allowed sunlight to enter through a clear-coated area for sunlight. The main purpose of this invention was to provide a novel method for overcoming the problems of drying in the open sun (United States patent).

In the following decades, solar drying technology has been the subject of research and improvements, both using natural and forced circulation, and with regard to heating from auxiliary sources [15,24,25]

Sun drying is still the most common method used to preserve agricultural products in many countries of the world: if advanced techniques are not available, farmers spread their products in thin layers on open ground or on mats where they are exposed to the sun and wind to be dried. Several authors report that significant losses can occur during natural sun drying mainly due to rodents, birds, insects, rain, storms and microorganisms [26]. It has been calculated that about 40% of food loss in less-developed countries derived from post-harvest handling including bad or incomplete drying practice. For this reason, in many of these countries many organizations and researchers are developing and comparing drying systems to obtain better performances in the food drying process from both an economic [27] and nutraceutical point of view.

## 3. Solar Cooker and Dryer: Classifications

**Figure 1:**
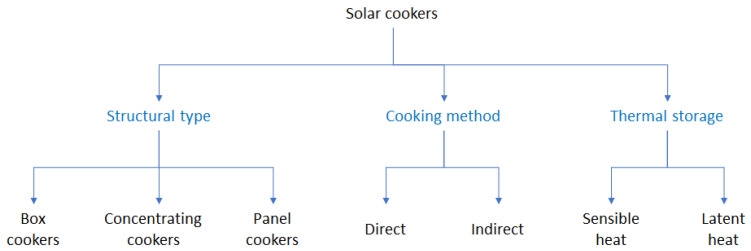
A general classification of solar cookers [28].

Here, we clarify the concept of solar cookers because this term can include different types of this general concept. We may refer to some classifications such as the one in Aramesh (Figure 1) [28].

**Figure 2:**
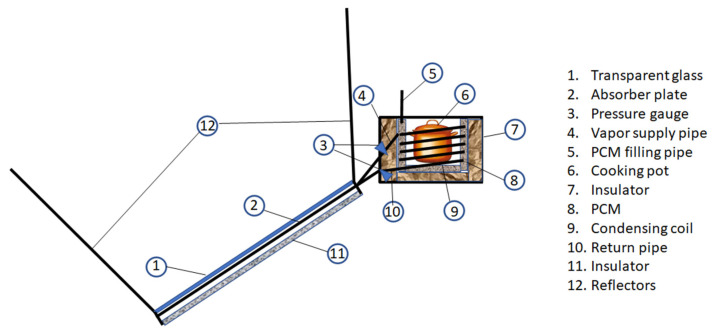
Solar cooker (SC) with indirect cooking method. A fluid with high thermal capacity is heated by the sun and transfers heat to the pot. By absorbing the heat PCM can prolong the cooking time with low sun irradiation.

This classification is based on how thermal energy from the sun is transferred to the cooking vessel. It is important to clarify that in this context the “cooking method” is “indirect” when the sun’s energy is captured by a part of the system and then transferred to the cooking box by means of specific fluid having a high thermal capacity (Figure 2).

**Figure 3:**
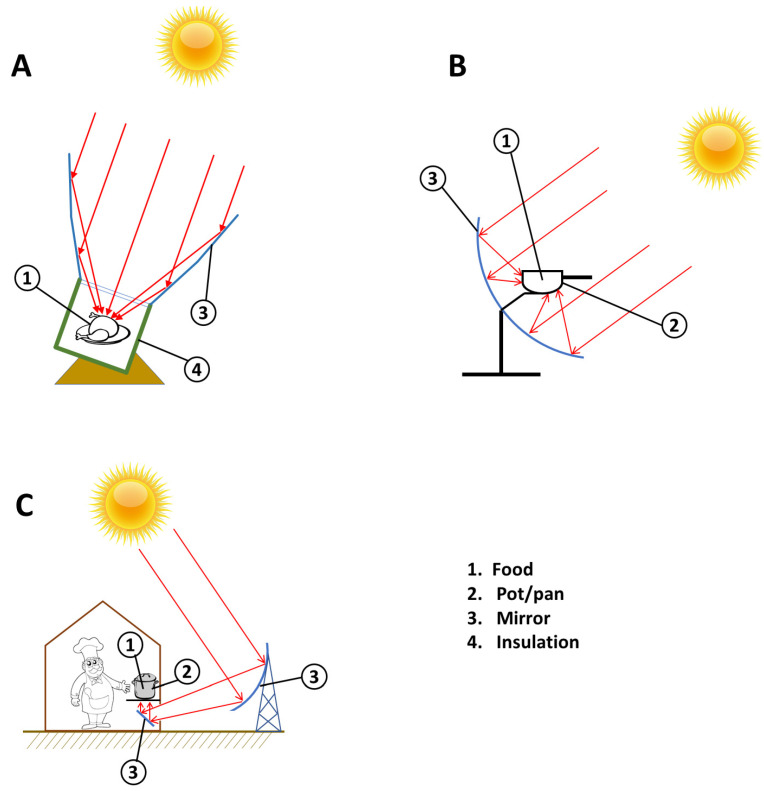
Sketched structures of Solar cookers (SCs) with direct cooking method. (**A**) SC with sunlight concentrated into the food; (**B**) SC with the focus of a parabolic sunlight absorber under the pot; (**C**) SC with a parabolic sunlight absorber that concentrates the heat under the stovetop by a system of reflecting mirrors.

However, since we are focusing on food transformation, we are more interested here on how energy is transferred to the food during the cooking process. Here, we may distinguish two different types of energy transfer, and both of them use the concentration by mirrors:

1. indirect transfer of the sun’s energy to the food (Figure 3B,C). Here we will always have at least a pot or pan or stovetop in the middle. Mirrors take the form of a parabola and the receiver is in the focus. Energy is concentrated on the pot and then the pot will soon transfer it to the food. With this approach the cooking will depend only on the temperature of the pot and the food will have the same treatment as in conventional cooking with the heat source placed below the pot.
2. direct light into the food. With this approach the sunlight is concentrated into the food, and the cooking process depends either on sunlight or heat (Figure 3A).
  Within type (1) it is important to underline the advantages for the approach, as in Figure 3C where the sunlight is focused below a stovetop, through a second reflection. The position of the focus is far from the parabola and the receiver is completely separated from the capturing system. This approach gives two advantages: (a) we can increase the size of the capturing system, and so the power of the oven; (b) to follow the sun, it is possible to move the parabola which keeps the focus always below the stovetop. Movement of the parabola can be automated, as has been suggested by Wolfgang Scheffler [29].

With regard to type (2), the approach based on sunlight concentrated into the food (Figure 3A), it is also important to note that the food is cooked with a strong contribution of the visible light, so in this case the wavelength is different compared to irradiation used in standard ovens. Most SCs have also glass at the top of the box, where the light goes through because the glass (also called “float”) is transparent to visible, near-UV and near- infrared.

**Figure 4:**
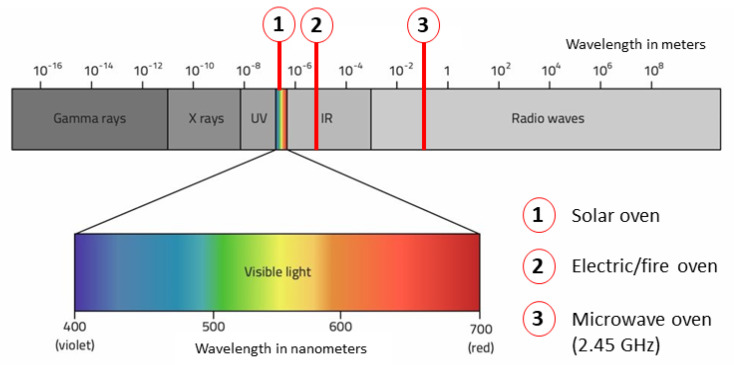
Wavelength ranges in oven cooking.

As we know, the microwave oven has a different way to cook compared to the standard oven heated by electricity or fire of standard fossil, as shown in Figure 4. As solar cookers are concerned, the sunlight wavelength range is close to IR but far from the microwave.

To compare the overall cooker efficiency between SCs is not an easy task, because this parameter is strongly dependent on the test conditions since it is given by the ratio between the amount of solar energy transferred to the test load and the pot and the solar energy being collected by the cooker. The value generally spams from 10 to 30%. In a recent paper, the overall cooking efficiency for Funnel solar cookers was calculated by Apaolaza-Pagoaga et al. [30] with values spanning from 10.2 to 11.8%. These values can be considered as very good results for low-cost cookers. Higher efficiencies were found by El-Sebaii et al. [31] where a box-type cooker was tested. The box cooker was able to cook most kinds of food with an overall utilization efficiency of 26.7%. Recently, a SC with a high-performance light-concentrating lens was tested [32]. Overall efficiencies were found to be from 7 to 11% when silicone oil was the load and from 11 to 26% when water was the load.

**Figure 5:**
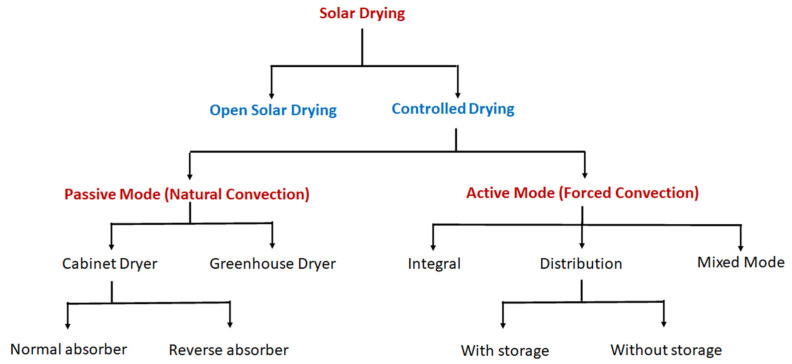
A general classification of solar dryers.

SDs (see their classification in Figure 5) are similar to SCs, but with two main differences:

1. maximum temperature is around 60 °C [33];
2. there is good ventilation to remove humidity from the air in the cabinet during the drying process.

**Figure 6:**
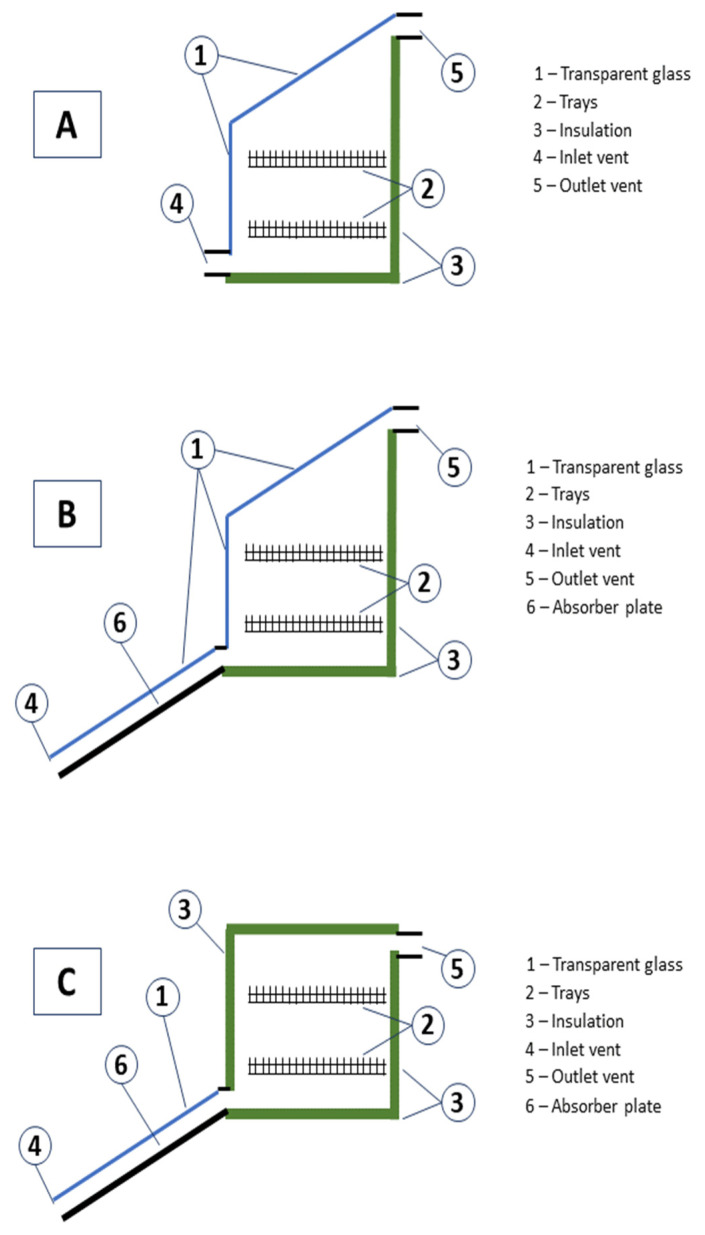
Sketched structure of different solar dryers (SDs).(**A**) Simple SD containing trays heated by direct sunlight; (**B**) SD with external air heating pipes; (**C**) SD with no direct sunlight on the food and external air heating pipes.

The SDs can be different in structure (Figure 6) but, in general, they allow food drying without problems of bad weather, pollution, or insects contamination. They should be easy to build, even in rural zones, and have low cost of usage [34]. In Figure 5 the “Controlled Drying” branch includes all these devices. A SD can be simply built as a greenhouse dryer (solar tunnel) in which sun rays selectively penetrate through glasses or transparent plastic polyethylene foils, or as a cabinet with many trays inside a rigid wood or metal structure closed by glass or transparent plastic (passive mode, Figure 5). Air circulation through perforated zones can eliminate moisture by internal convection (Figure 6A). In a more performant version external pipes further heat air by sunlight before letting it enter the cabinet (Figure 6B). In other cases, the cabinet has no direct penetration of sun rays on the food and sun is only needed to heat the air or produce energy by an absorber plate (Figure 6C). Lastly, hybrid sun dryers can use both sunlight and electricity (photovoltaic or from other sources) to heat air to enhance the drying process (mixed mode Figure 5) [27]. Natural or forced air flow permits the elimination of the major part of fruit and vegetables moisture.

## 4. Developments in Technology: Heat Storage

Many SC configurations are widespread and the search for improved performance has continued over the years but has recently become much more pronounced. The classification of SCs is therefore a rather complicated task (Figure 1) [35]. As discussed above, today most SCs without thermal storage generally fall into two main groups based on how heat is transferred to the cooking unit: direct and not direct, when heat is collected directly for cooking or via a fluid, respectively.

Typical examples of direct solar cookers are box and concentrating models. In the box-type, transparent glass covers a well-insulated box while multiple reflectors generally help to direct the sun rays towards the box (Figure 3A). Concentrating SCs, on the other hand, are based on optical principles that allow solar energy to be concentrated on the base of a pan or pot without intermediate obstructions, allowing it to reach very high temperatures (Figure 3B).

Not-direct SCs, conversely, are somewhat more elaborate devices as they comprise a collector and a cooking unit. The collector gathers the thermal energy, while heat exchange between the cooking unit and the collector is achieved by means of an intermediate transfer fluid (Figure 3C).

In the case of SDs, due to their high utilization in rural zones, their structure is generally simple as already presented (Figure 6) even if their use in food industry is increasing [24].

As SCs and SDs are based on sunlight, their main limitation is that they cannot be used, or lose performance, when the intensity of sunlight is low or even absent. Furthermore, another important limitation is related to the typically rather long cooking times, which could expose the user to significant solar radiation. For SDs the process can be prolonged over time, even for a few days. For this reason, there are temperature fluctuations, during night hours, that can slow down the drying process. To avoid these drawbacks, thermal energy storage (TES) is generally considered to be the best option. Among the commonly adopted techniques to store thermal energy, three main categories can be pointed out: heat storage can be achieved thermochemically, by sensible and latent heat exchange [36,37]. Since thermochemical storage is characterized by a rather low controllability, which makes its use difficult, this storage method will not be discussed here.

### 4.1. Sensible Heat Storage

Sensible storage materials can be found in both liquid and solid states. In order to compare them, it is necessary to estimate their thermal conductivities, heat capacities and densities. Among liquids, vegetable oils (coconut [38] and sunflower) and mineral oils (Mobiltherm [39], Shell Thermia C and Shell Thermia B) have been studied and compared in recent articles, with sunflower oil [40] providing the best performance.

Among solids, particular attention was paid to cast iron and granite [41], while two-phase oil-pebble beds were also studied [42,43].

As a general rule, in case of sensible heat storage the amount of heat stored is generally rather low [44,45]. It can be stated that solid sensible heat storage materials offered better energy density and thermal diffusivity than their liquid counterparts.

### 4.2. Latent Heat Storage

Materials involved in storage using latent heat exchange are called Phase Change Materials (PCM). In other words, PCM are materials whose phase change is used to store and release heat, and according to recent studies they are able to charge approximately 5 to 13 times more thermal energy per unit mass than materials in which only sensible heat is stored [46,47]. When a given temperature is reached, which varies from material to material, the absorption of solar heat from the environment brings to a phase transition of the material from solid to liquid. When the temperature drops, e.g., because of clouds or in the evening, the material solidifies and releases the stored heat. The stored thermal energy can then be used to extend the cooking process.

Even if all physical stages of phase transitions are in principle suitable as a PCM, the phase transitions involving gas phases are generally not exploited for these applications because of large volumes or high pressures necessary to store heat. On the basis of the requirements that a PCM must meet to be considered as thermal storage, between the different phase transitions the solid-liquid appears to be the most interesting for these purposes.

The main requirements are:

- phase change temperatures should be in the operating temperature range of the specific application;
- the enthalpy of phase change should be as high as possible;
- the phase change conditions should be reproducible;
- specific heat and thermal conductivity should be as high as possible;
- the volume during phase transitions should show minimal variations.

In addition, other characteristics to be met should be that the material remains chemically stable during cycles, it has low cost and availability, and it should not be corrosive, toxic or flammable [48].

A phenomenon that is quite common in some PCM and should be avoided is supercooling, i.e., the release of latent heat stored during solidification to a temperature other than the melting temperature [49]. Generally, high nucleation rate helps in avoiding supercooling of the liquid phase.

In direct SCs, the PCM is placed in contact (typically under the absorbent plate) [35] and heats the food in the cooking unit by conduction and convection while it gradually solidifies. It has been verified that the heat diffusion process between the PCM and the cooking vessel takes more time, particularly for evening cooking, because it occurs very slowly [50].

In not-direct SCs, the pan is connected to a collector via pipes and during sunshine hours, the solar radiation falls on an absorber (Figure 2). The heat transfer fluid passes through the absorber gathering the heat and transferring it to the PCM. Finally, this heat will be stored by the PCM and released to the cooking unit. During the day, the heat transferred from the PCM is used for cooking purposes. The stored heat is used for cooking purposes during the evening periods [51].

In SDs the PCM can be put in a latent heat storage tank external to the drying chamber [52] or in a flat plate collector connected on the top of a drying system structurally analogous to that represented in Figure 6B [53]. The energetic performances of various PCM solar dryers are extensively analyzed in the recent review of Mofijur [47].

The PCM usually used are organic, although inorganic PCM also exist. For organic materials, hydrocarbons, lipids and sugar alcohols are commonly adopted in solar applications while, among inorganic materials, salt hydrates and metallics are also broadly adopted. In particular, erythritol [54,55], acetanilide [51,56], acetamide [57] and stearic acid [58,59] are the most frequently used organic PCM in the experimental tests. Inorganic compounds include magnesium nitrate hexahydrate (MNHH) [59,60] and binary nitrate salt KNO_3_-NaNO_3_ [61], or ternary [62].

Comparing organic with inorganic PCM, it can be said that organic materials generally show larger latent heat, even if inorganic have higher heat capacities, densities and thermal conductivities, when comparable. Another important difference lies in the melting temperatures, which are generally lower than 120 °C for organic materials. However, it has to be considered that PCM analyzed for solar cookers are only a small part of available materials.

A wide range of potential PCM is presented by [63,64]. Furthermore, since cooking food generally requires temperatures above 100 °C, PCM with high phase change temperature are necessary. For this reason, the research on appropriate high-temperature PCM for solar cooker applications is very active, also bearing in mind that the cooking power is greatly influenced by thermal diffusivity of the storage medium and TES design.

When comparing sensible and latent heat storages, it is evident that latent heat storage produces smaller temperature changes than sensible heat but higher energy storage capacity and lower energy losses during phase change. Moreover, the exploitation of this storage mode generally allows good control [44,65,66,67]. For all these reasons, latent heat storage is the most exploited thermal storage technique in solar cookers.

As a final comment, it can be stated that, when possible, the best option is the integrated action of both thermal storage methods: while the stored latent heat helps in cooking, the sensible heat substance assists the PCM in its performances.

## 5. Processed Food by Sun: Ovens and Dryers

Cooking using a bonfire, or even dry dung, is a less expensive and routine way of cooking in poor countries, while charcoal barbecue is still very appreciated in the USA and Europe. These kinds of cooking methods enhance the production of heterocyclic aromatic amines (HAA) and Polycyclic Aromatic Hydrocarbons (PAH) that are extremely dangerous toxicants with mutagenic activity [68]. The use of a SC could be a significant improvement in the health of billions of poor people and in environmental protection while, in western rich countries, it could be a substitute option for barbecues to obtain healthier food in summer parties or in calamities where gas or oil is temporarily inaccessible. Unfortunately, the dissemination of SCs is connected with their adjustment to the social and cooking requirements of the people that use them. The necessity of a multilevel approach (economic, social, cultural and political) seems the only way of success [69]. In a report sponsored by the European Union, the use of solar cooking in Lebanon has been extensively scrutinized from an engineering, practical, social and economic point of view by Touma [70]. The study listed the opportunities that a SC could bring to poor families and even refugee communities.

The simplest SC “Cookit” (www.solarcookers.org (accessed on 7 October 2020)) can be made of cardboard covered by aluminum foil, folded so that the sun converges on a pot closed in a transparent plastic bag. These very inexpensive devices (around 5 USD) can reach temperatures up to 135 °C that permit water and food pasteurization along with food cooking. Touma reported the information present in the manual of this simple SC. Due to the low temperature reached, cooking time for a meal of 2 kg can vary from 1 to 8 h depending on the food (Table 1, Entry 1). With these cooking times, this kind of SC is considered a help to everyday meal preparation but, where it is used, it has not completely eliminated traditional cooking methods. It can be useful for cooking rice, boiling eggs, or preparing potatoes together with various dishes that do not need too much time for cooking. With this aim, it can be used not only in rural households but also in hospitals and-hostels of more sunny countries [34] or in campsites or outdoor lunches.

The countries in which the SCs have been most widely introduced are those with large periods of dry sunny days, so the majority of the literature is derived by researchers from equatorial countries. A “combined solar baking oven” was developed by Mekonnen et al. [71] to solve the cooking problems of Ethiopian people still largely accustomed to biomass fuel use in food preparation. The heating is obtained by sun rays directed onto the oven either from mirrors around or from one parabolic mirror under the cooking zone. A rectangular tray, easily extractable, was used to put the bread in to cook. With this device, the bread’s baking time lowers to 50 min, fast enough to be utilized in everyday preparations without a long period of attendance of the person under the sun (Table 1, Entry 2).

As a matter of fact, cooking with less expensive direct solar devices needs to check the position of the oven during the preparation and this often brings to the necessity for the cook to stay near the oven, under the sun during the hottest hours of the day. To address this inconvenience, Singh [72] has developed an indirect solar cooking system suitable for indoor cooking using a heater plate connected with an external parabolic collector. The energetic analysis was combined with the cooking of common use food. During the cooking experiments the maximum temperature reached was 109 °C and the cooking time of dishes were reasonable, spanning from the 45 min of Maggi (noodles) to 90 min of pulses (Table 2, Entry 3).

As we have shown, a drawback of the SCs is its difficulty in keeping the temperature high (180–250 °C) during cooking time regardless of solar radiation. In a long cooking process the maintenance of a stable and high temperature is important for the homogeneous cooking of the dish. In Nicaragua, the use of the SC to cook tortillas, a traditional and widely used dish, has proved to be ineffective due to the difficulty of keeping the temperature high enough during a relatively long time to obtain a good preparation [74,75].

For these reasons, the introduction of PCM in the SC design, to assure the conclusion of the long cooking processes, even without the presence of sunlight, is a great stimulus for its diffusion. Bhave and Kale [73] developed a solar storage cooking device formed by a special pot that could be heated thanks to the presence of an outer PCM layer heated by solar energy. The system involves a separate outdoor point of absorption where the PCM made of a mixture of NaNO_3_/KNO_3_ (60:40) salts, can absorb heat from a sun parabolic collector. Afterwards, the pot is delivered to the cooking point permitting to cook inside the kitchen. The system was tested by cooking potatoes and rice (Table 1, Entry 4). Two batches of rice (125 g each) cooked in boiling water (200 mL) were cooked in 20 + 20 min. Furthermore, 250 g of potatoes were fried in peanut oil in 17 min, at almost constant temperature (150–170 °C) with very good results. Normally, the impossibility of frying by SC, due to the need for high constant temperatures, was considered a drawback to its employment due to the cooking habits in many countries [70]. With the device developed by Bhave and Kale, the problem seems partially solved.

In more wealthy countries the use of the SC is more connected with environmental and social concerns or chefs’ experimentation. Many internet sites present recipes specifically designed for SCs and famous chefs take up the challenge of cooking delicious dishes using this alternative way of cooking.

However, even if in these last decades a great impulse has been devoted to the improvement of the technical performances of SCs to permit a broad use of these devices, few research groups have investigated the impact of the solar cooking on the flavor, taste, and nutraceutical properties of food so prepared. This is partially because many SCs are used as a traditional oven, to cook food only by heat irradiation. In these cases, the effects of baking are similar to those of a traditional oven, when used at analogous temperatures.

The heating of food at a high temperature—generally 180–250 °C—triggers loss of water, denaturation of proteins, gelation of collagen and depolymerization of complex sugars. The main visible effects are the changes in texture and colour, together with the generation of the aroma and flavour derived by the Maillard reaction between proteins and carbohydrates [76]. This is especially pleasant in roasted meat due to the increase of tenderness, with consequent ease of chewing and digestion. Obviously, many other effects are less appreciable such as a faster oxidation of lipids, vitamins and carotenoids’ degradation, antioxidants loss [77] and the production of various derivatives such as hydroxymethylfurfural (HMF) and acrylamide [68].

Generally, every kind of cooking method produces a variation in vitamin content, even considering the weight change due to water loss. For instance, in the case of the hydrosoluble vitamin C, research has demonstrated that the higher the quantity of water used in cooking (boiling, blanching, steaming and microwaving) the higher the loss detected. In this case, microwave cooking can almost completely retain vitamin C content [78]. On the other hand, during the cooking process vitamin E is retained more in green vegetables than in roots, while beta-carotene content is completely preserved or even enhanced by the partial disruption of its complexes with proteins [79].

The use of direct solar irradiation on food together with heating, could further modify food nutraceutical properties. UV light is helpful in preventing bacterial spoilage and fungal infection [80] but it can activate photolytic radical reactions that can further decrease the content of antioxidants, vitamins and other nutraceuticals that are fundamental for the beneficial impact of food on our diet. A few studies have analyzed the effect of direct solar irradiation on cooked food; however, many research groups have studied the effect of the sun on food, seeds and vegetables dried using SDs and these studies can give us an idea of the possible effects of UV light in direct solar cooking.

In many developing countries SDs are more and more used for the production of dried local fruits, vegetables and spices. Drying procedures were often traditional food treatments used to avoid microbial and fungal attacks on harvested products. The food, dehydrated to a safe extent, has a longer storage time and can be more easily used and exported, contributing to healthier lives for farmers and economic development [27]. Various drying processes analyzed in this paper are reported in Table 2 with their final moisture content and dehydration time. For instance, a SD can be filled with up to 50 Kg load of wheat flour or with up to 4 kg of curry leaves and the drying process may reach the required water content in 4–6 h (0% moisture in flour, and up to 4% in curry leaves) (Table 2 Entry 1) [12]. As SDs are the most used devices for the processing of food by the sun, many research groups have analyzed different drying methods (natural solar desiccation, SD, electric oven, etc.) to compare nutritional and sensory qualities, sometimes also considering the economic sustainability of the technique by the farmers [89].

Mohammed et al. compared the traditional systems of desiccation of mango and pineapple with an improved SD, a solar cabinet connected with a series of empty tubes that enhance air heating by sun (see Figure 6B) [81]. This SD enhanced the organoleptic attributes; for instance, an increase of sugars content (in mango: 14.3 ± 0.3 vs. 9.1 ± 0.3 mg/100 g) and mineral content (Ca, Zn, P, Fe, Mn and Cu) of the dried fruits (Table 2 Entry 2) with respect to fresh fruits. Furthermore, even if all drying processes resulted in a lowering of the total phenolic, Vitamin C and Vitamin A, in comparison with fresh fruit, the improved SD produced minor losses than open solar drying. So, compared with traditional processes, in mango dried with this improved SD the total phenolic content (TPC) proved to be 0.75 ± 0.03 vs. 0.44 ± 0.06 g/100 g; Vit C 35.6 ± 0.4 vs. 27.5 ± 0.4 mg/100 g and Vit A 849 ± 6 vs. 820 ± 4 mg/100 g [81].

Hamid et al. [82] studied the effect of different drying methods (natural sun, solar tunnel, mechanical cabinet, oven, lyophilization) on the antioxidant activity of various part of pomegranate fruit (aril, pomace, flavedo and albedo) to be utilized in pharmaceutical and nutraceutical industry (Table 2 Entry 3). TPC and total flavonoid content (TFC), together with metal chelating content, were generally lowered by all the drying processes that use hot air in comparison with low temperature lyophilization. In the pomegranate, direct sunlight produces the higher losses of polyphenols (TPC aril: 1.54 ± 0.01 mgGAE·g^−1^; pomace: 0.34 ± 0.01 mgGAE·g^−1^) and flavonoid (TFC aril: 40.92 ± 0.08 mgQuE·100 g^−1^; pomace: 10.94 ± 0.61 mgQuE·100 g^−1^), but the simple use of transparent polyethylene coverage, as in a solar tunnel dryer, partially reduces the antioxidant loss (TPC aril: 1.60 ± 0.01 mgGAE·g^−1^; pomace:0.36 ± 0.02 mgGAE·g^−1^; TFC: aril: 44.00 ± 0.045 mgQuE·100 g^−1^; pomace: 13.50 ± 0.17 mgQuE·100 g^−1^). This evidence confirms the negative effect of UV rays on fruit processing.

Many studies have evidenced that fruits suffer loss of nutraceuticals directly depending on the heating temperature and time of drying process [12]. In general, the higher the temperature and the time of the drying process, the higher the loss of antioxidants and vitamins [81]. Similar studies on eggplant drying processes confirm that the TPC, beta carotene contents and antioxidant capacity were affected by all kinds of desiccation methods as a function of the temperature utilized [90]. It must be kept in mind that a too rapid desiccation should be avoided because it could bring to incomplete elimination of moisture in the core of fruit pieces and this could produce storage and health problems.

Vangdal et al. [83] have also verified that not all different plum cultivars behave in the same way if exposed to various drying processes (Table 2 Entry 4). In a comparison between conventional and organic plums, the anthocyanins, neo-chlorogenic acid (NCA) and ascorbic acid contents turned out to be different after oven drying (OD), SD or freeze drying (FD). If the FD always demonstrated to be the technique that preserves the higher quantity of antioxidants and vitamins, in comparison, the level of NCA and the Folin Ciocalteu index were less depleted by OD, while SD produces a minor loss of anthocyanins and Vit C, above all in Jubileum Cultivar plums.

The data of Deus et al. [33] goes against the general trend just highlighted: in the comparison of different cocoa beans drying methods, the antioxidant activity, together with the phenolic and methylxanthine content, were better conserved by the traditional open-air sun drying method. In particular, catechins (0.02 ± 0.00 vs. 0.037 ± 0.00 mg·g^−1^), epicatechins (0.09 ± 0.05 vs. 1.037 ± 0.02 mg·g^−1^) together with caffeine (1.60 ± 0.06 vs. 2.33 ± 0.02 mg·g^−1^) and theobromine (11.14 ± 0.59 vs. 14.96 ± 0.55 mg·g^−1^) were all less concentrated after the drying process using a solar cabinet (Table 2, Entry 5) instead of traditional drying. Furthermore, while the antioxidant activity measured with the Oxygen Radical Absorbance Capacity (ORAC, 611 ± 35 vs. 618 ± 72 μmolTE·g^−1^) and the Ferric Reducing Antioxidant Power (FRAP, 375 ± 2 vs. 378 ± 2 μmol Fe^++^·g^−1^) showed a difference under the limit of detection, the DPPH (94.09 ± 0.02 vs. 94.85 ± 0.05 %S.R.L), the β-carotene-linoleic acid assays (32.33 ± 0.03 vs. 32.71 ± 0.04 %I.O.) confirmed the lower antioxidant depletion by traditional exposure to direct sun. This data could be due to the different temperatures of seeds drying processes. Generally, direct sun drying works at lower temperatures than artificial dryers and, for this reason, the loss of antioxidants and alkaloids could be reduced by using traditional methods. Furthermore, the cocoa seeds could be less sensitive to the UV light than fruits due to the stronger structure of tegument in comparison with fruit peel.

Many desiccation methods were also compared to find more eco-friendly and economic techniques for the preparation of flours, dry leaves or spices useful in the food industry. In the study of Kiharason et al. the nutrient integrity of pumpkin flour dehydrated using the SD (Table 2 Entry 6) was compared with the electric oven or open sun drying. All desiccation processes have enhanced the content of beta-carotene (fresh fruit: 16.6 ug/g; dried flour: 74.84 ug/g) and proteins (fresh fruit: 2.6%; dried flour 13.8–16.5%) while lowered the level of Calcium and Iron by half, and of Zinc by a fifth, if compared to the fresh fruit. Interestingly, direct solar drying produced a lower concentration in beta-carotenes in comparison to solar cabinets because of the partial photo-degradation of carotenoids [84].

On the other hand, in the Stevia R. leaves drying process using SD with natural (Table 2 Entry 7) or forced air flow with or without mesh protection, the analysis of color change evidenced that the temperature is more crucial than the UV exposure [85]. Analogously, lemongrass dried with SD (50.6 °C, Table 2, Entry 8), showed a greater variation of color and pH (6.25 vs. 5.9) with respect to direct sun drying (34.7 °C) probably depending on the higher thermal stress suffered [86].

In addition, spices have been studied to compare the effect of OD with SD processes. In the case of hihatsumodoki fruits (Piper retrofractum Vahl), an Asiatic pepper, the higher content of piperine (21.7 ± 3.2 mg/gdm) was obtained by solar drying (41.9 °C, Table 2 entry 9) while, antioxidants resulted better preserved by oven drying at 60 °C instead of the SD as evidenced by the data of Total Phenolic Content (SD vs. OD = 18.5 ± 3.8 vs. 15.2 ± 2.2 mg GAE/gdm) [87]. Similarly, ginger powder obtained by desiccation under shade at room temperature overtook either sun (Table 2 Entry 10) or oven drying. In particular, when using the SD the moisture content was better lowered (3.5 ± 0.08% instead 3.8 ± 0.08%) but the beta-carotene (0.68 ± 0.02 vs. 0.81 ± 0.01 mg/100 g DM) and ascorbic acid (2.2 ± 0.08 vs. 3.8 ± 0.07 mg/100 g DM) content were lost the most [88].

As we have presented, research has either investigated the cooking time of foods to analyze the energy performance of the various SCs or focused attention on the variations of nutraceuticals only in SDs. To the best of our knowledge, no general studies are available on the activity of direct sunlight on cooked food. Certainly, this lack should be covered in order to have a deep and complete knowledge of the potential and drawbacks of these devices.

## 6. Perspectives

Today, the usage of solar cookers is not widespread enough globally. According to our knowledge it is present in a few areas of the developing countries or in refugee camps thanks to humanitarian associations while in Europe and the USA SCs are seldom used in camping areas or by amateur groups. The development of SCs has two different aims: (i) to be low cost and durable to help poor people to avoid fuel shortages and (ii) to keep the quality high and ease the cooking process with a device without continuous movement of the oven.

From an engineering perspective there are several improvements to help achieve even more quality results with the cooking process. As for the first aim, many researchers are studying low cost solar cookers that could withstand use more than the simple panel model in comparison with other cooking systems [91].

As for the second aim, at the moment it is not possible to regulate the temperature correctly. This is a limit experienced by skilled chefs who started to use the solar cooker in several worldly events. Having in mind the Sheffler SC model, it is possible, from the technical point of view, to automate the mirrors in order to concentrate the sun by moving each of them, or a group of them, according to the instantaneous temperature in the oven. In this way the oven could maintain a constant temperature and the cook can focus only on his job.

About the use of thermal energy storage systems in SCs and SDs, it is important to point out that PCM shows both positive aspects and drawbacks. In fact, a thermal storage such as PCM can prevent temperature gradients by imposing, during the cooking or drying procedures, the melting temperature of the material which is supposed to remain constant during the phase change process. By identifying the proper material and increasing its thermal performances by some triggering processes, cooking times could be generally extended by up to 3–4 times, if compared to SCs with no thermal storage, while the drying process can be shortened in the same way. Moreover, the appropriate choice of PCM and, consequently, its melting temperature, is equivalent to setting the cooking temperature of the food, which is an advantage for the cook who does not have to waste time managing this parameter as well.

On the other hand, inserting a thermal storage implies inserting an additional load that could delay the cooking time. In addition, as thermal storage, it should be chosen a material that does not damage the food in case of leakages. With this in mind, sugar alcohols, which can be obtained from fruit and plants, would seem to be particularly appropriate.

## 7. Conclusions

In conclusion, this review reports a large overview about SCs and SDs applied to the processing of food. The explored aspects range from the technical description of SCs and SDs, to the possibility to store the heat produced by solar irradiation and then use it for time-consuming cooking or drying procedures exploiting PCM, to the analysis of the composition of cooked and dried food, with special attention to the nutraceuticals content after drying process. To summarize the main results of this study, we would like to highlight some strong points and issues posed by the analyzed literature, that are also useful to open challenging perspectives for future studies in the field.

Concerning the effect of SCs and SDs on food, no complete and exhaustive research is available on the effects on food of solar cooking while the studies on solar drying have evidenced an heterogeneous behavior depending on the type of processed food, direct or indirect irradiation, drying temperature and time. Reported data confirm the effect of direct UV rays in depletion of vitamins and antioxidants in fruits and vegetables. In some cases, it has been reported that the use of polyethylene foils that let only specific radiations through, has overcome or at least reduced this problem.

While plenty of literature has analyzed the effect on nutraceuticals of sun-dried food, the identification of nutritional benefits of food cooked using direct sun radiation has not been yet analyzed and this could be a boost to the use of SCs in the more developed countries where a healthy lifestyle is increasingly considered.

It certainly appears essential to carry out further research in this field in order to deepen the knowledge on the effect of the various radiations on nutraceuticals even in the solar cooking of food and eventually to find solutions that can selectively let penetrate only harmless wavelengths. The final aim is to obtain not only tasty but healthy foods with solar cookers, while looking at the environmental sustainability.

## Figures and Tables

**Table 1:** Cooking time with different solar cooker.

Food	Cooking Time (min)	Max Temp (°C)	Solar Cooker Used	Reference
rice	60–120	135	Cookit (solarcookers.com)	[70]
potatoes	180–240			
beans, lentils	180–240			
large roast meat	300–480			
bread	180–240			
vegetables	60–120			
fish	60–120			
bread	50	121	combined solar baking oven	[71]
noodles (Maggi)	45	109	SC with externat rays collector	[72]
rice	65			
potatoes	70			
dal (pulse)	90			
rice	20	150–170	PCM solar cooker device	[73]
fried peanuts	17			

**Table 2:** Drying process on various food using solar dryers.

Food	Weight	Final Moisture Content	Drying Time (h)	Max Temp (°C)	Reference
weath flour	50 kg	0%	4	60	[12]
curry leaves	4 kg	0%	6		
mango bar (2 layers)	7 kg/m^3^	<12%	16		
fig bar (2 layers)	7 kg/m^3^	<12%	16		
mango slices	7 kg/m^3^	<8%	16		
apple	9 kg/m^3^	<8%	10	59	
sapota	4 kg/m^3^	<8%	8	50	
papaya	9 kg/m^3^	<8%	18	42	
mango slices	a	<3%	9	40.3	[81]
pinapple slices	a	<5%	9.5		
pomegranade (arils)	2 kg	12%	75	36	[82]
pomegranade (albedo)	0.5 kg	10%	45	37	
plum	7 kg/m^2^	0%	192	45	[83]
cocoa beans	a	8.80%	168	<60	[33]
pumpkin	a	12.80%	13.3	<60 ^a^	[84]
stevia leaves	1 g	7.80%	3.5	53	[85]
lemongrass	50 g	5.00%	8	50.6	[86]
hihatsumodoki fruit	1.74 g	26.10% ^b^	24	41.9 ^c^	[87]
ginger powder	a	3.50 ± 0.08%	a	a	[88]

^a^: not determined; ^b^: relative mass after drying; ^c^: average temperature.
